# Influence of Embedding Microcapsules on Tribological Properties of Alumina Ceramics Prepared by Gel Casting

**DOI:** 10.3390/ma18092110

**Published:** 2025-05-04

**Authors:** Ze Sun, Hui Chen, Xianglong Meng, Guangchun Xiao, Zhaoqiang Chen, Mingdong Yi, Jingjie Zhang, Wenyu Liu, Chonghai Xu

**Affiliations:** 1 School of Mechanical Engineering, Qilu University of Technology (Shandong Academy of Sciences), Jinan 250353, China; 17865276368@163.com (Z.S.); xgc@qlu.edu.cn (G.X.); czq@qlu.edu.cn (Z.C.); 15053103340@163.com (M.Y.); zjj@qlu.edu.cn (J.Z.); wenyuliu@qlu.edu.cn (W.L.); xch@qlu.edu.cn (C.X.); 2 Shandong Institute of Mechanical Design and Research, Jinan 250031, China; mengxl_jn@163.com

**Keywords:** embedded solid lubrication, gel casting, alumina, oil-containing microcapsules

## Abstract

The continuous advancement of technology has led to escalating demands for superior tribological performance in industrial applications, necessitating the enhancement of ceramic materials’ frictional properties through innovative approaches. Solid-lubricant embedding is a widely employed lubrication strategy in metals. However, the challenge of machining holes on ceramic surfaces remains a significant barrier to applying this lubrication technique to ceramics. Gel casting, as a near-net-shaping process, offers several advantages, including uniform green body density, low organic content, and the capability to fabricate components with complex geometries, making it a promising solution for addressing these challenges. In this study, alumina ceramics with small surface holes designed for embedding oil-containing microcapsules were fabricated via gel casting using an N-hydroxy methylacrylamide gel system, which demonstrates lower toxicity compared to conventional acrylamide systems. The fabricated alumina ceramic materials exhibited a high density of 98.2%, a hardness of 16 GPa, and a bending strength of 276 MPa. The oil-containing microcapsules were self-synthesized using hexafluorophosphate ionic liquid as the core material and polyurea-formaldehyde as the wall material. The research results show that under conditions of using an alumina ball, sliding speed of 10 cm/min, load of 5 N, and at room temperature, the material with a microcapsule content of 15 wt% and embedded hole diameter of 1.2 mm reduced the friction coefficient from 0.696 in an unlubricated condition to 0.317. Moreover, the embedding of microcapsules further improved the wear resistance of the alumina.

## 1. Introduction

Ceramic materials demonstrate high hardness, excellent wear resistance, superior heat resistance, as well as exceptional corrosion and oxidation resistance, making them widely used in fields such as aviation, space, military, automotive, and electronic industries [1]. Nevertheless, under dry sliding conditions, the friction coefficient of 0.5–0.8 of ceramic materials reveals potential opportunities for tribological performance enhancement through material design innovations [2]. Introducing self-lubricating phases into ceramic composites is a widely employed strategy for friction coefficient reduction and wear resistance improvement in tribological applications [3,4]. Yang et al. [5] fabricated Al_2_O_3_/TiC ceramic composites with 10 vol% CaF_2_ addition through co-sintering. Tribological testing under 20 N normal load revealed that the CaF_2_ incorporation effectively reduced the friction coefficient from 0.65 to 0.5, attributed to the formation of a solid lubricating film at the contact interface. The incorporation of solid lubricants in ceramic matrices frequently leads to degradation of mechanical properties [6,7]. Recent advances employ core-shell structured lubricants, where protective coatings encapsulate lubricant particles, thereby preserving the ceramic’s structural integrity while retaining lubricating functionality. Zhang et al. [8] demonstrated that incorporating Al_2_O_3_-coated hexagonal boron nitride (h-BN) into Si_2_N_3_ ceramics significantly enhanced mechanical properties compared to uncoated h-BN additions. The study reported a 27.0% improvement in hardness, 5.9% increase in fracture toughness, and 22.2% enhancement in flexural strength, attributed to the protective alumina coating maintaining matrix continuity.

Embedded solid lubrication is a technique where solid lubricants are incorporated into pre-fabricated holes on the material’s surface to enhance its lubrication properties [9]. During friction, the lubricants are gradually released from the holes, effectively reducing the material’s friction coefficient. This lubrication method has been widely applied to copper alloys and bearing steel materials. However, due to the high hardness of ceramic materials, it is difficult to be machined. Machining the holes necessary for embedded solid lubricants on ceramics presents significant challenges, which has long impeded the application of this technology in ceramic materials.

Gel casting is a near-net-shaping process for components pioneered by the Oak Ridge National Laboratory [10]. Compared with the other ceramic material preparation processes, gel casting offers several advantages, including low organic content, uniform green body density, high green body strength, and the ability to near-net-shape large and complex components. Currently, this process has been extensively used in the research and industrial production of ceramic materials [11,12]. Therefore, it can be inferred that gel casting technology provides a simple and feasible method for fabricating ceramic components with pre-embedded holes designed for solid lubricant incorporation. Gel casting slurries can be incorporated into water-based and non-water-based systems based on the solvent used. Among these, water-based slurries have been more widely adopted due to their environmental friendliness and cost-effectiveness [13]. In water-based slurries, the acrylamide (AM) system exhibits excellent gelation properties and good green body strength after gelation, making it the most widely used system in gel casting processes [14,15]. However, AM is a neurotoxin that can harm the health of operators [16]. Huang et al. [17] successfully fabricated an alumina turbine rotor using a methacrylamide (MAM) gel casting system. Similar systems are the N methylol acrylamide system, N-hydroxy-methacrylamide (NMAM) system [18], N,N-dimethylacrylamide (DMAA) [19], and so on. They demonstrate comparable gelation behavior to the AM system while exhibiting significantly reduced toxicity [20]. Isobutene-maleic anhydride copolymer (ISOBAM) has recently been identified as a non-toxic gel casting system [21,22]. Since ISOBAM itself possesses dispersing capabilities, it can replace or reduce the need for additional dispersing agents [23]. However, the current ISOBAM system faces the challenge of producing green bodies with relatively low mechanical strength. In addition to these systems, researchers have also explored gel casting using epoxy resins [24] and agar [25] starch [26], carrageenan [27], and sodium alginate [28] materials, making notable advancements in the field. Among these systems, the NMAM system offers advantages such as low toxicity, high solid content, and high green body strength, making it highly promising for applications in embedded solid lubrication.

Microcapsules are core-shell-structured micro-containers formed by encapsulating solid, liquid, or gas core materials with natural or synthetic polymers as wall materials. Currently, commonly used methods for preparing microcapsules include in situ polymerization [29,30], interfacial polymerization [31], solvent evaporation [32], physical impregnation [33], and sol-gel processes [34]. Many researchers have explored microcapsules encapsulating lubricating oil within wall materials, achieving significant improvements in lubrication performance [35,36,37]. Compared to solid lubricants such as graphite, molybdenum disulfide, boron nitride, and PTFE, which are commonly used in embedded solid lubrication to form solid lubrication films on friction surfaces, microcapsules offer the advantage of forming a more effective liquid lubricating film. This liquid film further reduces the friction coefficient and significantly enhances the tribological performance of the material [38].

A literature review reveals no existing reports on embedding oil-containing microcapsules into ceramic surfaces to improve their lubrication properties. We believe this method is an effective approach to improve ceramic lubrication performance, benefiting from the microcapsules’ ability to generate liquid lubricating films during friction. In this study, alumina ceramics with surface small holes were fabricated using the gel casting method. Self-synthesized oil-containing microcapsules, utilizing hexafluorophosphate ionic liquid as the core material and polyurea-formaldehyde as the wall material, were embedded into the surface holes of the alumina. The lubrication performance of the resulting material was analyzed. We hope this study broadens the application of microcapsules in ceramic materials and provides a new way for achieving self-lubricating ceramics.

## 2. Materials and Methods

### 2.1. Materials and Reagents

The average grain size of alumina powder (Shanghai Chaowei Nano Technology Co., Ltd., Shanghai, China) is 500 nm. The reagents used in the gel casting process are detailed in Table 1. Deionized water was used as the solvent, with N-hydroxy methacrylamide (NAMA, CAS 924-42-5) and N,N-methylene bisacrylamide (MBAM, CAS 127-19-5) serving as the monomer and crosslinking agent, respectively. Polyacrylic ammonium (PAA, CAS 9003-03-6) was used as the dispersant, n-octanol (CAS 111-87-5) as the defoaming agent, ammonium persulfate (CAS 7727-54-0), and tetramethylethylenediamine (CAS 110-18-9) as initiator and catalyst. Epoxy resin (CAS 1675-54-3, Pinyi Metallographic Technology Co., Ltd., Guangzhou, China) was used as the binder for microcapsules.

**Figure 1:**
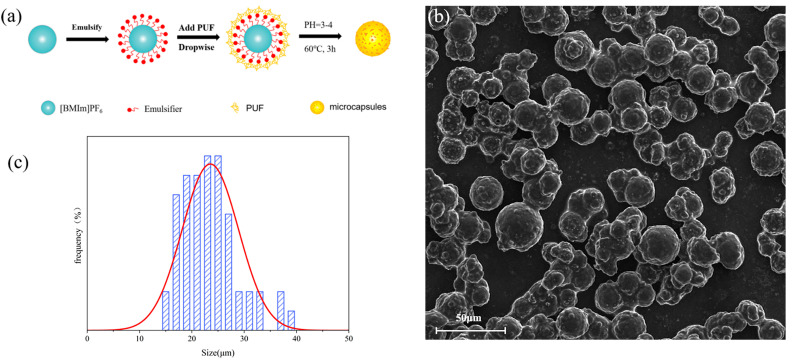
(**a**) Synthesis process of the microcapsules, characterization of prepared microcapsules: (**b**) scanning electron microscope (SEM) image, (**c**) grain size distribution image.

The oil-containing microcapsules were self-synthesized in a laboratory. The core material consists of hexafluorophosphate ionic liquid (CAS 16919-18-9, Shanghai Maclin Biochemical Technology Co., Ltd., Shanghai, China), which has good lubrication properties, while the wall material is polyurea-formaldehyde (PUF, CAS 9011-05-6, Shanghai Maclin Biochemical Technology Co., Ltd.). The synthesis process is illustrated in Figure 1a. Initially, the ionic liquid is emulsified to form spherical particles. Subsequently, PUF is gradually added while adjusting the pH and temperature. This facilitates the adsorption of PUF onto the surface of the ionic liquid, resulting in the formation of the desired microcapsules. As revealed in Figure 1b, the microcapsules fabricated via this approach exhibit well-defined spherical structures with a high production yield and impurity-free characteristics, validating the effectiveness of the synthesis protocol. The particle size distribution of the microcapsules is presented in Figure 1c, indicating an average diameter of approximately 25 μm. The lubricant content within the microcapsules was determined using an acetone extraction method. This involved crushing the microcapsules, washing them multiple times with acetone, and subsequently drying them. The lubricant content was calculated by comparing the weight of the microcapsules before and after the cleaning process, yielding a lubricant content of 68%.

### 2.2. Gel Casting of Alumina Ceramics

**Figure 2:**
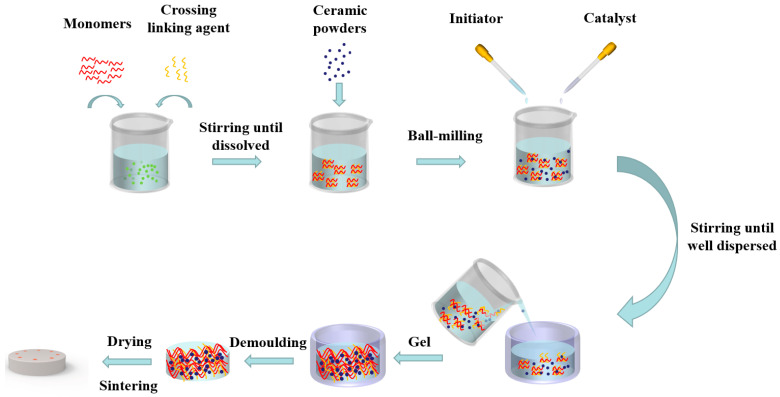
Schematic representation of the gel casting process towards the fabrication of dense ceramics.

The schematic representation of the gel casting process is shown in Figure 2. First, the monomer AM (0.5 wt%), crosslinking agent MBAM (AM:MBAM = 10:1), and dispersant PAA were incorporated into deionized water and stirred until dissolved to obtain a premixed solution. Ammonia and a hydrochloric acid solution were used to adjust the pH value of the premixed solution. The ceramic powder was added to the premixed solution in the required mass fraction to obtain the ceramic slurry. A suitable amount of n-octanol was then added to the slurry to reduce bubble formation. After ball milling the slurry for 24 h, ammonium persulfate (0.2 wt%, based on alumina powder) was introduced and the mixture was vacuum agitated for 30 min. Then, tetramethylethylenediamine (0.02 wt%, based on alumina powder) was introduced and the mixture was vacuum agitated for another 15 min. The slurry was poured into a 3D-printed resin mold and subjected to vacuum gelation at 60 °C for 20 min. The vacuum pressure used was 0.02 MPa. During this process, the monomer and crosslinking agent undergo a chain reaction to form a three-dimensional network within the material, thereby fixing the alumina powder and resulting in a green body with certain strength. Following demolding, to prevent the sample surface from peeling off during the drying process, the sample was first dehydrated at room temperature for 24 h, followed by 12 h at 40 °C, and finally for 12 h at 60 °C. The sample was sintered in a muffle furnace under an air atmosphere. During sintering, the temperature was first raised to 600 °C at a heating rate of 2 °C/min and maintained for 1 h to remove organics. The temperature was then raised to 1500 °C at a rate of 2 °C/min and maintained for 2 h, followed by furnace cooling. Epoxy resin was used as the binder to embed the self-synthesized microcapsules into the holes on surface of the sintered alumina ceramic.

### 2.3. Characterization

**Figure 3:**
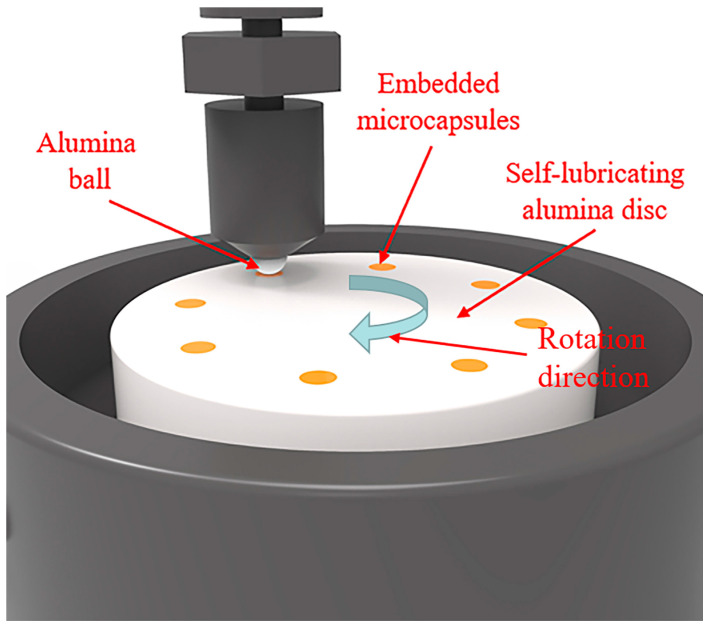
Schematic of the tribological test.

The pH value of the alumina suspension (0.1 wt%) was measured using a Zeta potential analyzer (Zetasizer Nano, Malvern, Worcestershire, UK) to determine the optimal dispersion pH of the alumina suspension. The viscosity of the ceramic slurry with different dispersant content and solid content was measured using a material dynamic properties tester (MCR302 Anton Paar, Graz, Austria). The binder removal temperature of the green body was determined using a thermogravimetric analyzer (TGAQ50, TA Instruments, New Castle, DE, USA). The surface morphology and elemental analysis of the material were performed using a scanning electron microscope (Phenom Prox, Phenom Scientific, Eindhoven, The Netherlands). The Archimedes method was applied to measure the density of the sintered alumina. A vacuum drying oven (DZF-6050, Shanghai Jinghong Experimental Equipment Co., Ltd., Shanghai, China), was used to meet the requirements of vacuum stirring and vacuum gelation. The hardness and flexural strength of the material were tested using a microhardness tester (HXD-1000, Shanghai Taiming Optical Instrument Co., Ltd., Shanghai, China) and a universal testing machine (AGX-X5KN, Shimadzu Corporation, Tokyo, Japan), respectively. The bending strength was tested on bar-shaped specimens with dimensions of 4 mm (width) × 3 mm (height), using a span length of 20 mm. Hardness testing was performed under a load of 196 Kgf. To guarantee testing reliability, each group underwent five repeated measurements for both bending strength and hardness, and the mean value was adopted as the test result. Tribological tests were conducted using a friction and wear analyzer (THT800, Anton Paar, Graz, Austria). The schematic of tribological test is shown in Figure 3. The tribological pair consists of a commercially available alumina ball with a diameter of 6 mm and a self-prepared alumina disc with ionic liquid-containing microcapsules embedded on its surface. The alumina disc is fixed at the bottom of the instrument and the alumina ball at the top contacts the circumferential surface of the disc containing microcapsules. The tribological properties of the materials were evaluated under constant sliding speed (10 cm/min) at room temperature, with systematic variations in (1) microcapsule content (0–20 wt%), (2) embedded hole diameter (0.8 mm–1.2 mm), and (3) applied normal load (1–10 N). The sampling rate of tribological tests is 50 Hz. The average friction coefficient for the tribological tests was calculated by summing all the acquired data points and dividing by the total number of measurements. To ensure statistical reliability, all tribological tests were performed in triplicate to reduce the impact of random errors on the data. When the friction curves from three repeated tests showed good consistency, a representative curve was selected to present the experimental results under this condition. The three-dimensional morphology of the wear track after the friction and wear test was analyzed using a 3D optical microscope (Contour Elite K, Bruker, San Jose, CA, USA).

## 3. Results and Discussion

### 3.1. Pourability of Ceramic Slurry

**Figure 4:**
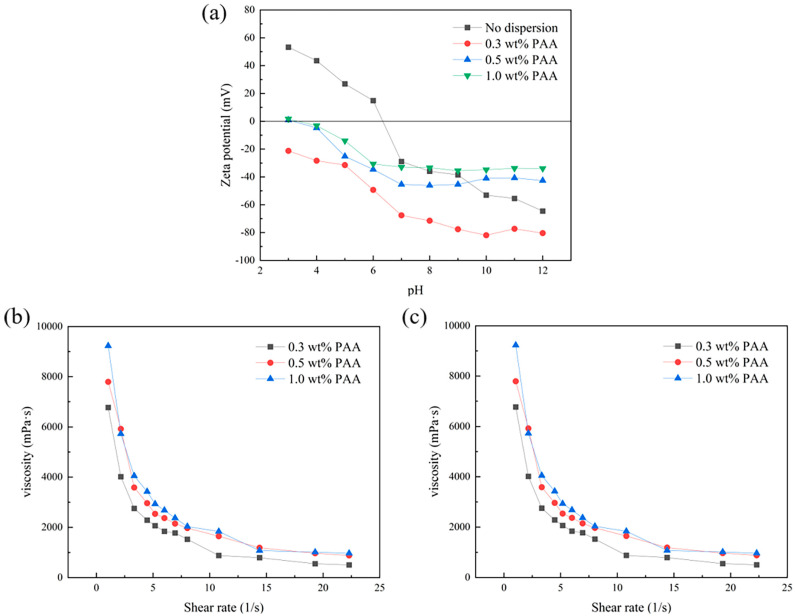
Effect of dispersant on zeta potential (**a**) and effect of dispersant (**b**) and solid content (**c**) on the viscosity of alumina slurry.

The proper dispersion of ceramic powders is essential for preparing slurries with a high alumina content and good fluidity, which is the premise of the gel casting method for producing high-performance, dense alumina. The dispersion of alumina powders is primarily governed by the electrostatic repulsion between particle surfaces, with zeta potential being the key parameter for characterizing this repulsion. The zeta potential of the slurry under varying pH conditions was determined with a Zeta potential analyzer Each sample was measured three times and the average value was used as the final result. As shown in Figure 4a, the zeta potential of alumina powder in deionized water varies with pH, reaching a maximum of −64.5 mV at pH 12. The introduction of dispersants modifies the surface charge density or alter the electrical double layers, thereby enhancing the material’s dispersion properties. For instance, with 0.3 wt% PAA, the zeta potential reaches −80.4 mV at pH 10, surpassing the −64.5 mV observed without dispersant. However, an excessive amount of dispersant can lead to oversaturation and excessive adsorption, reducing the zeta potential of alumina particles. With an increase in PAA content to 0.5 wt% and 1.0 wt%, the maximum zeta potential values decrease to −46.1 mV and −35.5 mV, respectively. This trend aligns with the viscosity changes observed in the ceramic slurry at different dispersant concentrations, as shown in Figure 4b. Excessive dispersant not only fails to reduce slurry viscosity but actually increases it. Therefore, the optimal casting performance of the alumina slurry is achieved with a PAA content of 0.3 wt% and a pH value of 10.

Alumina slurry with a higher solid content results in a smaller interparticle distance after gel casting, which indicates higher green body strength and better sintering densification. However, excessive solid content can negatively affect the slurry’s fluidity. As shown in Figure 4c, when the dispersant PAA content is 0.3 wt%, the viscosity of the alumina slurry gradually increases as the solid content rises from 50% to 54%. However, when the solid content continues to increase to 56%, the viscosity of the ceramic slurry rises sharply, reaching as high as 32,330 mPa·s at a shear rate of 1 s⁻¹. Since the ceramic slurry with a solid content of 54% maintains a relatively high solid content while still exhibiting good fluidity (with a viscosity of only 6768 mPa·s at a shear rate of 1 s⁻¹), we chose to use the slurry with a solid content of 54 wt% for casting.

### 3.2. Characterization of the Alumina

**Figure 5:**
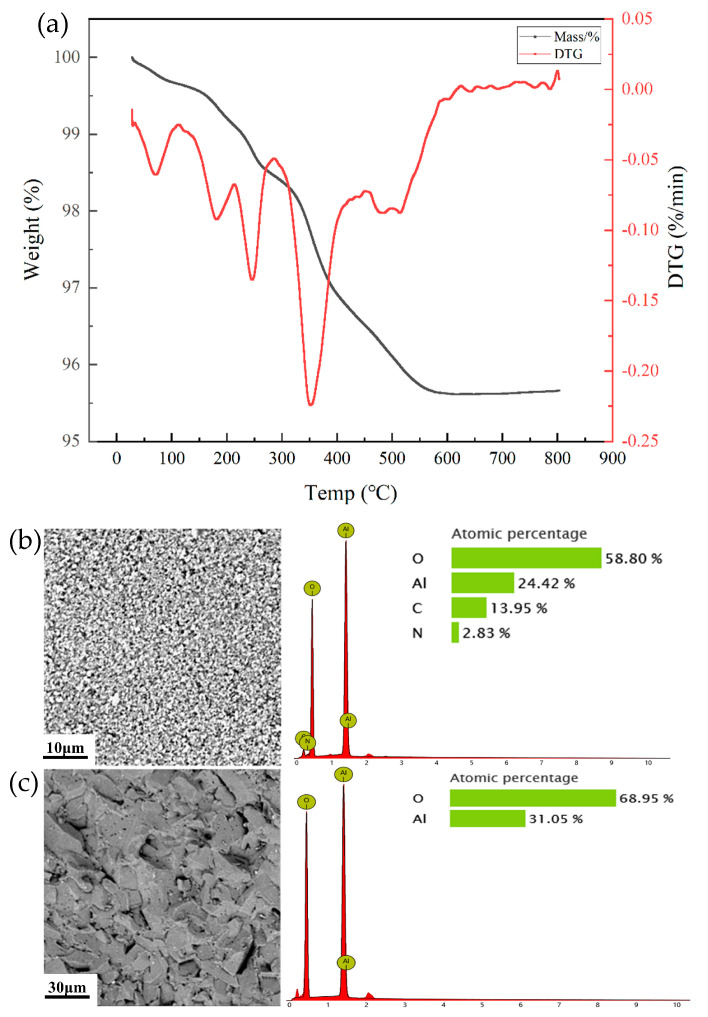
TGA of the gel casting green body (**a**), SEM images of the green body (**b**), and sintered sample (**c**) with the corresponding elemental analysis.

The slurry with a dispersant content of 0.3 wt% and a solid content of 54% underwent gelation, demolding, and drying, resulting in a high-strength green body with a flexural strength of 32.3 MPa. The scanning electron microscopy (SEM) image of the green body is shown in Figure 5b. From the image, it can be observed that the alumina particles are arranged relatively uniformly under the influence of the monomer and crosslinking agent.

During the sintering process, the monomer, crosslinking agent, and other organic components in the green body are converted into gases and expelled. However, if the heating rate is too rapid, the decomposition of organic components within the green body occurs too quickly, making it difficult for the gases to escape in time. The residual gases can lead to larger pores in the sintered body, which negatively affects the densification and performance of the material. To determine the decomposition temperature of the organic matter, thermogravimetric analysis (TGA) was performed on the green body from room temperature to 800 °C at a heating rate of 10 °C/min. The thermogravimetric curve of the green body is shown in Figure 5a. Below 300 °C, the mass loss is primarily due to the removal of residual moisture. As the temperature reaches around 300 °C, the mass decreases significantly as the organics begin to decompose and it stabilizes at 600 °C when the organic materials have fully decomposed, with no further mass loss.

During the organics removal process, the green body was initially raised to 600 °C at a rate of 2 °C/min and maintained for 1 h, ensuring the complete expulsion of organics. The temperature was then increased to 1500 °C to continue the sintering process. Elemental analysis of the green body before and after sintering shows that the C and N elements, which make up the organic material, largely disappear after sintering. Only the Al and O elements, which constitute alumina, remain in the material. It proves that the organics are fully decomposed during sintering. Furthermore, as shown in Figure 5c, the sintered material exhibits good densification and the binder removal has not resulted in noticeable pores within the material.

Through measurements, the radial shrinkage of the sample was found to be 21.9%, the axial shrinkage 22.0%, and the shrinkage of the surface hole diameter 21%, indicating relatively uniform shrinkage. The density of the material is 98.2%, with a hardness of 16 GPa and a flexural strength of 276 MPa. These mechanical properties are comparable to those of dense alumina ceramics prepared by traditional powder metallurgy.

**Figure 6:**
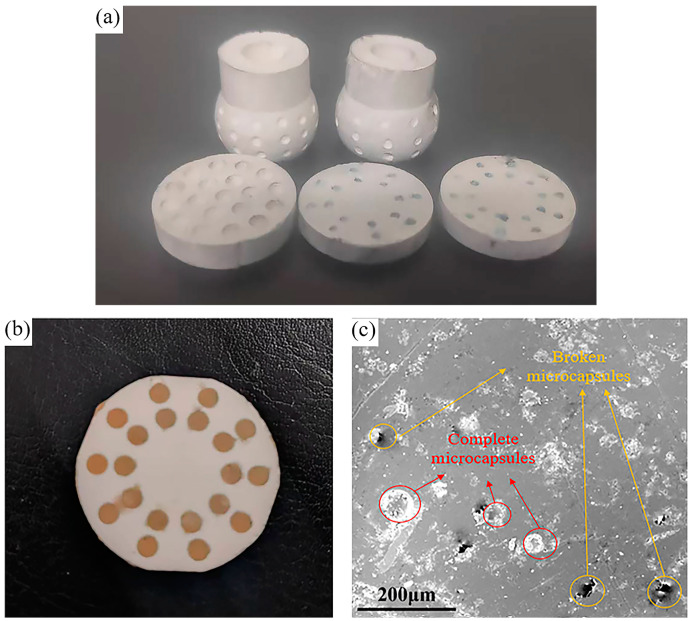
Parts prepared by gel casting (**a**), alumina disc embedded with ionic liquid-containing microcapsules (**b**), and SEM image of the disc surface (**c**).

Figure 6a illustrates the discs and spherical sliding bearings with embedded holes manufactured using the gel casting technique. After sintering, the alumina ceramic disc was polished using a grinding machine and diamond polishing paste. Epoxy resin was used as a binder to embed the microcapsules into holes on the surface of the ceramic disc. Excess microcapsules on the ceramic surface were removed using sandpaper, followed by surface polishing with a polishing machine. The alumina disc embedded with ionic liquid-containing microcapsules on its surface was successfully obtained, as shown in Figure 6b. Figure 6c presents the SEM image of the embedded material, where broken microcapsules from the polishing process and complete microcapsules distributed on the surface can be clearly observed.

### 3.3. The Tribological Properties of the Material

**Figure 7:**
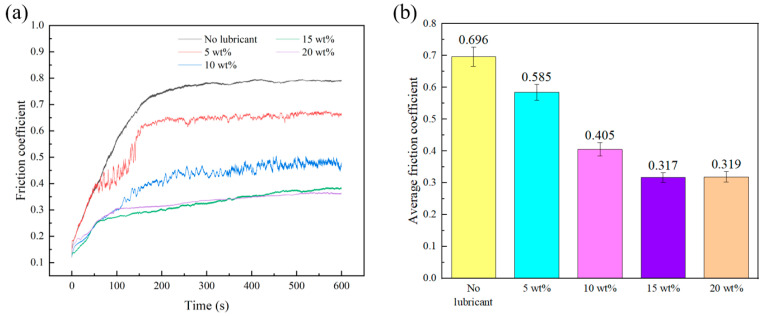
Influence of microcapsule contents on friction coefficient curves (**a**) and average friction coefficient (**b**).

Pure alumina and alumina ceramic discs with embedded holes of 1.2 mm diameter and microcapsule contents of 5 wt%, 10 wt%, 15 wt%, and 20 wt% in the embedded part were fabricated to explore the impact of different microcapsule contents on the frictional properties of the alumina discs. Figure 7a shows the friction coefficient curve of the sample under a load of 5 N and a speed of 10 cm/min. Due to the polishing treatment, the alumina disc has a low surface roughness, resulting in a relatively low initial friction coefficient. As the friction time increases, the friction coefficient of all samples gradually increases until reaching a stable stage. Compared to the average friction coefficient of 0.696 for the pure alumina sample, the embedding of microcapsules effectively reduced the friction coefficient of the material. As the microcapsule content increased, the friction coefficient showed a decreasing trend. When the microcapsule content reached 15 wt%, the average friction coefficient of the sample decreased to 0.317, representing a 54.5% reduction. The embedded microcapsule lubrication strategy provides a novel approach for fabricating self-lubricating alumina with a low friction coefficient. And compared with the average friction coefficient (0.4–0.6) of self-lubricating alumina sintered with traditional solid lubricants (e.g., CaF_2_ [5] or graphene [39]), embedding microcapsule alumina demonstrates superior tribological performance due to the formation of a continuous liquid lubricating film at the friction interface.

**Figure 8:**
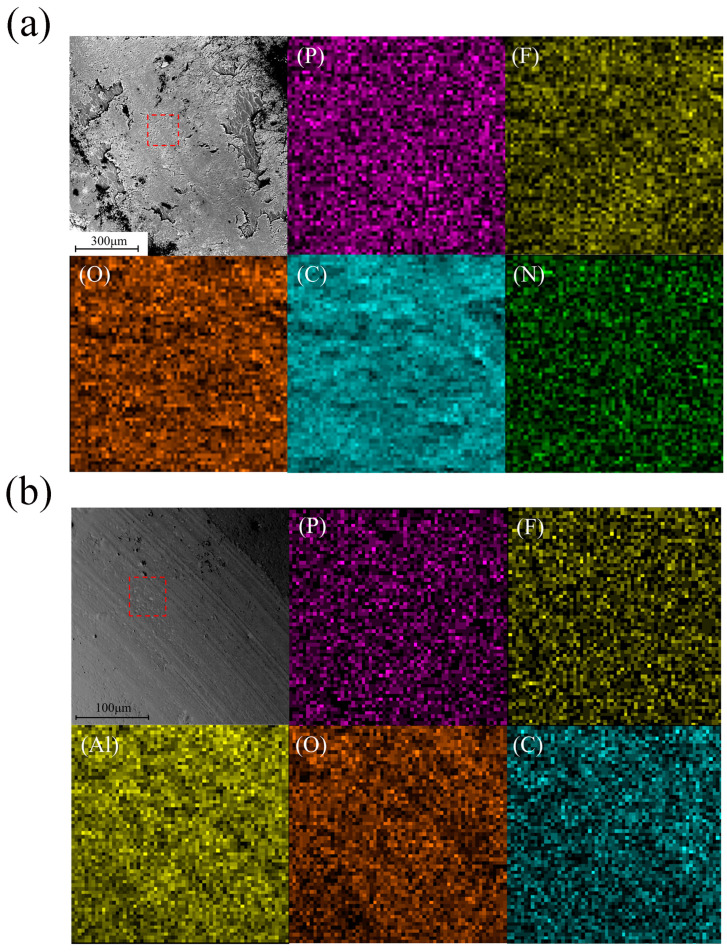
EDS analysis of the embedded (**a**) and non-embedded (**b**) regions within the wear track (dashed box) on the surface of the alumina disc embedded with 15 wt% microcapsules.

Figure 8 shows the energy dispersive spectrometer (EDS) of the wear area on the surface of the alumina disc embedded with 15 wt% microcapsules. It can be observed that both the embedded and non-embedded regions exhibit a uniform distribution of P and F elements across the entire surface. This indicates that during the friction process, the embedded part wears down, the microcapsules are broken, and the hexafluorophosphate ionic liquid inside the microcapsules is released onto the friction surface, thereby reducing the friction coefficient of the material. At the same time, this also indicates that during the friction process, 15 wt% microcapsules are sufficient to form a uniform lubricating film on the friction surface. Therefore, when the microcapsule content increases to 20 wt%, the friction coefficient is similar to that of the 15 wt% microcapsule sample. In consideration of economic factors, the optimal content of microcapsules is 15 wt%.

**Figure 9:**
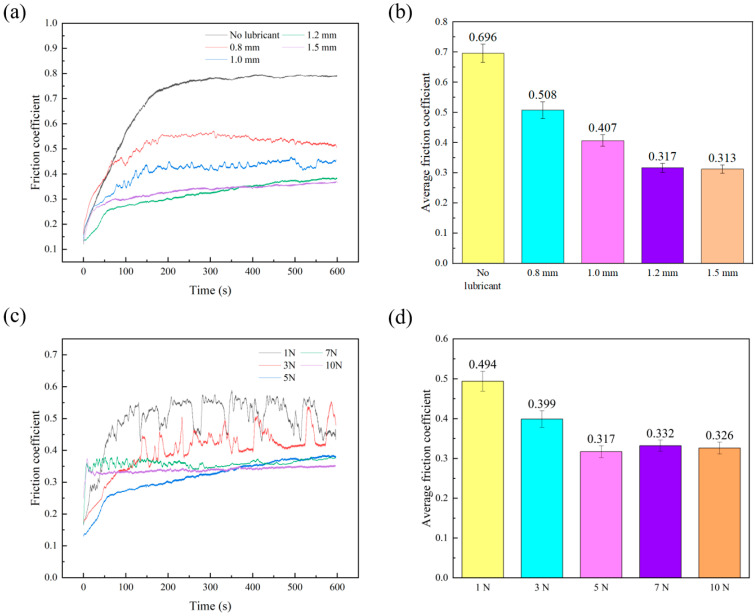
Influence of the diameter of the embedded hole (**a**,**b**) and load (**c**,**d**) on friction coefficient curves and average friction coefficient.

The friction curves measured for alumina discs with 15 wt% microcapsules, under a load of 5 N and a friction speed of 10 cm/min, with varying embedded hole diameters (10 evenly distributed holes along the 8 mm circumference of the alumina disc), are shown in Figure 9a. The friction curves consistently exhibit an initial increase, followed by gradual stabilization. A larger embedded hole diameter leads to the release of more microcapsules onto the friction surface, promoting the formation of a liquid lubricating film. Consequently, as the embedded hole diameter increases from 0.8 mm to 1.2 mm, the friction coefficient of the sample progressively decreases. When the embedded hole diameter reaches 1.2 mm, a uniform liquid lubricating film forms effectively during friction, and further increases in hole diameter do not significantly affect the friction coefficient. However, the mechanical properties of the embedded material are weaker compared to the alumina matrix. An excessively large hole diameter reduces the proportion of ceramic material on the friction surface, negatively impacting the overall performance of the material. Based on these considerations, an embedded hole diameter of 1.2 mm is determined to be optimal for the prepared alumina discs.

Meanwhile, we also tested the impact of different loads on the frictional properties. As shown in Figure 9c, when the applied load is less than 3 N, the compressive force exerted on the microcapsules is insufficient to rupture them, hindering the effective release of the ionic liquid. Consequently, the friction coefficient remains relatively high and exhibits noticeable fluctuations. Specifically, under a load of 1 N, the friction coefficient shows a distinct “high-low-high-low” pattern, which is likely due to the differing friction characteristics of the embedded and unembedded regions: the embedded regions are composed of resin, which has a lower friction coefficient, while the unembedded regions consist of alumina, which has a higher friction coefficient. As the load increases, a greater number of microcapsules rupture, facilitating the release of ionic liquid. This leads to a decrease in the friction coefficient, which gradually stabilizes as the lubrication effect becomes more uniform. When the load reached 5 N, the friction curve and average coefficient of friction stabilized without significant fluctuations. This indicates that a 5 N load is sufficient to form a uniform lubricating film on the material surface. Therefore, the embedded self-lubricating alumina developed in this study should ideally be applied under loads of 5 N or higher to achieve optimal lubrication performance.

**Figure 10:**
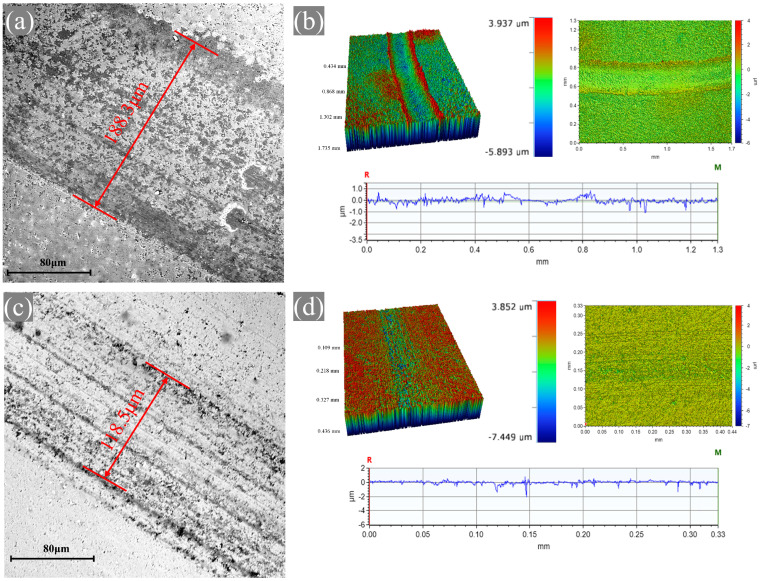
SEM images and 3D morphology of wear tracks for pure alumina discs (**a**,**b**) and embedded microcapsule alumina discs (**c**,**d**).

The wear morphology of the embedded microcapsule alumina and pure alumina discs was observed after extending the friction test to 1 h under a load of 10 N and a sliding speed of 10 cm/min, with an embedded hole diameter of 1.2 mm and a microcapsule content of 15 wt%, as shown in Figure 10. Compared to the pure alumina disc, which exhibited a wear scar width of 188.3 μm, the alumina disc with embedded lubrication showed a significantly reduced wear scar width of only 115.5 μm, representing a 37% decrease. This indicates that the microcapsules effectively reduced the wear of the alumina balls, thereby decreasing the wear scar width on the ceramic disks with embedded lubrication. From the three-dimensional morphology of the wear scars, it can be observed that despite the excellent wear resistance of alumina, shallow wear scars still appeared on the surface of the pure alumina disc. In contrast, the surface of the embedded microcapsule alumina exhibited only slight wear with almost no depth. This demonstrates that the embedded microcapsule lubrication not only forms a liquid lubricating film to reduce the friction coefficient of alumina but also significantly decreases its wear.

Owing to its demonstrated low friction coefficient and reduced wear rate, the embedding microcapsule alumina shows significant application potential in low-friction components, particularly alumina spherical plain bearings.

## 4. Conclusions

Alumina ceramics with uniform shrinkage and small surface holes were prepared using low-toxicity gel casting and pressureless sintering. By embedding ionic liquid-containing microcapsules into the surface holes using resin as a binder, embedded alumina ceramics were obtained and their lubrication properties were investigated. The main conclusions are as follows:

1. For the NMAM gel casting system, the optimal parameters for preparing alumina slurries were achieved with the addition of 0.3 wt% PAA dispersant, a pH value of 10, and a solid loading of 54%.
2. The prepared alumina ceramics achieved a relative density of 98.2%, a hardness of 16 GPa, and a flexural strength of 276 MPa after binder removal at 600 °C for 1 h and pressureless sintering at 1500 °C for 2 h. The mechanical properties of the materials prepared by gel casting are comparable to those of alumina ceramics prepared by traditional powder metallurgy.
3. Embedding ionic liquid-containing microcapsules into the surface holes significantly improved the tribological performance of alumina. When the embedded hole diameter was 1.2 mm and the microcapsule content was 15 wt%, the friction coefficient decreased by 54.5% under a 5 N load. At a 10 N load, the friction coefficient was comparable to that at 5 N. Compared to pure alumina, the embedding microcapsules alumina demonstrated a 37% reduction in wear track width along with significantly decreased wear depth. Embedding microcapsules effectively improve the tribological properties of alumina.

Compared to conventional sintered alumina with solid lubricants, the embedding microcapsule alumina achieves a significantly lower friction coefficient. This superior tribological performance demonstrates considerable application potential for low-friction components, particularly in alumina spherical plain bearings. Building on this work, we propose future studies to systematically investigate how different microcapsule compositions affect the tribological performance of embedded self-lubricating alumina, aiming to identify the optimal microcapsule formulation.

## Figures and Tables

**Table 1:** Reagents used in the gel casting process.

	Reagent Name	Chemical Formula	Purity	Manufacturer
Solvent	Deionized water	H_2_O	-	-
Monomer	NMAM	C_4_H_7_NO_2_	98%	Shanghai Aladdin Biochemical Technology Co., Ltd., Shanghai, China
Crosslinking agent	MBAM	C_7_H_10_N_2_O_2_	99%	Tianjin Xien Si Biochemical Technology Co., Ltd., Tianjin, China
Dispersant	PAA	C_3_H_7_NO_2_	40%	Shanghai Aladdin Biochemical Technology Co., Ltd., Shanghai, China
Initiator	Ammonium persulfate	(NH_4_)_2_S_2_O_8_	98.5%	Shanghai Yien Chemical Technology Co., Ltd., Shanghai, China
Catalyst	Tetramethylethylenediamine	C_6_H_16_N_2_	99%	Shanghai Yien Chemical Technology Co., Ltd., Shanghai, China

## Data Availability

The original contributions presented in this study are included in the article. Further inquiries can be directed to the corresponding author.

## Abbreviations

The following abbreviations are used in this manuscript:

AM	Acrylamide
DMAM	N, N-dimethylacrylamide
EDS	Energy dispersive spectrometer
h-NB	Hexagonal boron nitride
MAM	Methacrylamide
NMAM	N-hydroxy methacrylamide
PUF	Polyurea-formaldehyde
SEM	Scanning electron microscope
TGA	Thermogravimetric analysis
ISOBAM	Isobutene-maleic anhydride copolymer
